# Detection of Feline Coronavirus Membrane Gene Based on Conventional Revere Transcription-Polymerase Chain Reaction, Nested Reverse Transcription-Polymerase Chain Reaction, and Reverse Transcription-Quantitative Polymerase Chain Reaction: A Comparative Study

**DOI:** 10.3390/ijms26146861

**Published:** 2025-07-17

**Authors:** Chiraphat Kopduang, Witsanu Rapichai, Chalandhorn Leangcharoenpong, Piyamat Khamsingnok, Thanapol Puangmalee, Siriluk Ratanabunyong, Amonpun Rattanasrisomporn, Thanawat Khaoiam, Hieu Van Dong, Kiattawee Choowongkomol, Jatuporn Rattanasrisomporn

**Affiliations:** 1 Graduate Program in Animal Health and Biomedical Sciences, Faculty of Veterinary Medicine, Kasetsart University, Bangkok 10900, Thailand; chiraphat.kop@ku.th (C.K.); chalandhorn.le@ku.th (C.L.); piyamat.kha@ku.th (P.K.); thanapol.puan@ku.th (T.P.); 2 Department of Companion Animal Clinical Sciences, Faculty of Veterinary Medicine, Kasetsart University, Bangkok 10900, Thailand; tswitsanu@gmail.com (W.R.); thanawat.khao@ku.th (T.K.); dvhieuvet@vnua.edu.vn (H.V.D.); 3 Department of Biochemistry, Faculty of Science, Kasetsart University, Bangkok 10900, Thailand; ae.med@hotmail.com (S.R.); fsciktc@ku.ac.th (K.C.); 4 Interdisciplinary of Genetic Engineering and Bioinformatics, Graduate School, Kasetsart University, Bangkok 10900, Thailand; fgraapr@ku.ac.th; 5 Kasetsart University Veterinary Teaching Hospital, Faculty of Veterinary Medicine, Kamphaeng Saen Campus, Kasetsart University, Nakhon Pathom 73140, Thailand; 6 Faculty of Veterinary Medicine, Vietnam National University of Agriculture, Trau Quy Town, Gia Lam District, Hanoi 131000, Vietnam

**Keywords:** feline coronavirus, RT-PCR, RT-qPCR, nested RT-PCR, FIP

## Abstract

Feline coronavirus (FCoV) is a major pathogen causing feline infectious peritonitis (FIP), a lethal disease in cats, necessitating accurate diagnostic methods. This study developed and compared novel primers targeting the FCoV membrane (*M*) gene for enhanced detection. Specific primers were designed for the *M* gene and their performance evaluated using reverse transcription-PCR (RT-PCR), nested RT-PCR, and reverse transcription-quantitative PCR (RT-qPCR) on 80 clinical effusion samples from cats suspected of FIP. Specificity of assays was tested against other feline viruses, with sensitivity being assessed via serial dilutions of FCoV RNA. RT-qPCR had the highest sensitivity, detecting 9.14 × 10^1^ copies/µL, identifying 93.75% of positive samples, followed by nested RT-PCR (87.50%, 9.14 × 10^4^ copies/µL) and RT-PCR (61.25%, 9.14 × 10^6^ copies/µL). All assays had 100% specificity, with no cross-reactivity to other viruses. The nested RT-PCR and RT-qPCR outperformed RT-PCR significantly, with comparable diagnostic accuracy. The novel primers targeting the FCoV *M* gene, coupled with RT-qPCR, delivered unparalleled sensitivity and robust reliability for detecting FCoV in clinical settings. Nested RT-PCR was equally precise and amplified diagnostic confidence with its high performance. These cutting-edge assays should revolutionize FCoV detection, offering trusted tools that seamlessly integrate into veterinary practice, empowering clinicians to manage feline infectious peritonitis with unprecedented accuracy and speed.

## 1. Introduction

Feline coronavirus (FCoV), a member of the genus *Alphacoronavirus* within the order *Nidovirales*, is a significant pathogen in cats, causing feline infectious peritonitis (FIP), a leading cause of feline mortality [1]. Its single-stranded RNA genome, spanning 27–32 kb, encodes a replicase polyprotein, four structural proteins (spike (S), membrane (M), nucleocapsid (N), and envelope (E)), and non-structural accessory proteins (3a, 3b, 3c, 7a, and 7b) [2]. The S protein facilitates receptor binding and membrane fusion [3], the E protein contributes to viral envelope formation [4,5,6], the N protein supports RNA synthesis and virion assembly [7], while the roles of accessory proteins, particularly 3c, are linked to virulence and replication, with 7a and 7b modulating immune responses [8,9]. The *M* gene, encoding a type III transmembrane glycoprotein, is the most abundant structural protein and is critical for viral assembly and budding, interacting with the N, S, and E proteins [10]. The transition from benign feline enteric coronavirus (FECV) to virulent feline infectious peritonitis virus (FIPV) is driven by mutations in multiple genes, with the spike (*S*) gene playing a pivotal role in altering viral tropism and enabling systemic infection through monocyte and macrophage targeting [3,11,12]. Mutations in the membrane (*M*) gene have also been identified as contributors to this transition, influencing pathogenicity and immune evasion by inhibiting host interferon responses [11,12,13,14,15]. Additionally, accessory genes, such as 3c, 7a, and 7b, have been implicated in modulating virulence and replication efficiency [8,9,11]. Due to its conserved nature and specific mutations associated with FIPV virulence, the *M* gene serves as a promising target for diagnostic assays, complementing the roles of other genes in FCoV pathogenesis [15,16,17]. Its stability and high transcription levels during viral replication enhance its suitability for reliable molecular diagnostics, particularly for FIP, prompting its selection as the primary target in this study [18].

FCoV are classified into two pathotypes: FECV, which replicates in the enteric epithelium causing subclinical or mild enteritis, and FIPV, a lethal variant that targets monocytes and macrophages, spreading systemically due to FECV mutations [1,19]. The variable S proteins define two serotypes: Serotype I, which is dominant in 80–95% of cases with a unique spike protein, and Serotype II, resulting from recombination with canine coronavirus and transmissible gastroenteritis virus [2,20]. Both can present as FECV, confined to intestinal cells, or FIPV, causing fatal systemic infection within 1–3 weeks [21]. Spread via fecal–oral transmission, FCoV is highly contagious in multi-cat settings, such as shelters, with Serotype I shedding lasting 2–3 months (13% become carriers), and Serotype II shedding for two weeks (no carriers) [22,23]. Most cases are asymptomatic or mild; however, a few develop FIP with vasculitis, presenting as “wet” effusions or “dry” lesions, requiring precise diagnosis [24].

Several genes, including the *N*, *S*, and ORF1a/1b, have been utilized as targets for FCoV detection, each with distinct advantages. The *N* gene, highly expressed during viral replication, is frequently targeted for its abundance in clinical samples; however, its sensitivity can vary in early or low-viral-load infections [25]. Conversely, the *S* gene, with its high variability, is often used for differentiation assays to identify FIPV-specific mutations but may compromise primer binding across diverse strains due to sequence heterogeneity [11,26]. In contrast, the *M* gene offers a unique combination of high conservation, ensuring consistent primer binding across FCoV strains, and specific mutations associated with the FECV-to-FIPV transition, making it a robust target for both detection and diagnostic specificity [16,17]. Its critical role in viral assembly and pathogenicity further enhances its suitability for reliable molecular diagnostics, particularly for FIP, prompting its selection as the primary target in this study.

Diagnosing FIP involves a thorough evaluation of a cat’s history, clinical symptoms, and physical exam findings to guide the selection of diagnostic tests, which analyze samples, such as blood, effusions, tissues, cerebrospinal fluid, aqueous humor, and fine-needle aspirates, with effusion tests offering higher predictive value than blood tests [27,28]. Immunohistochemistry (IHC) remains the gold standard by detecting FCoV antigens in macrophage-associated lesions, though direct methods, such as PCR and quantitative RT-PCR (RT-qPCR), identify viral RNA with high sensitivity and specificity, while indirect methods, such as enzyme-linked immunosorbent assays (ELISA) and immunofluorescence assays (IFA), detect antibodies/antigen [27,29,30,31]. Real-time RT-PCR, widely used for nearly two decades, excels at detecting and semi-quantifying FCoV RNA in samples, such as effusions or feces, achieving up to 95% sensitivity and specificity when optimized; however, it requires proper sample collection, RNA preservation, inhibitor neutralization, and skilled execution [20,26,32]. Despite its precision and speed, false positives can occur in asymptomatic cats, necessitating cautious interpretation [33].

Our previous work introduced reverse transcription loop-mediated isothermal amplification (RT-LAMP) with a neutral red indicator as a rapid, sensitive, and specific method to detect FCoV, targeting the ORF1a/1b region at 58 °C in 50 min with a detection limit of 20 fg/µL and no cross-reactivity with other feline infections [34]. Recent work developed a colorimetric RT-LAMP assay with xylenol orange (XO) targeting the nucleocapsid (*N*) gene of FCoV, enabling rapid, specific detection at 65 °C in 60 min with a limit of detection of 1.7 × 10^1^ copies/µL and no cross-reactivity with other feline viruses. This assay achieved 100% sensitivity and specificity compared to qPCR, offering a simple, visual, and cost-effective alternative for diagnosing FCoV, the causative agent of FIP, despite being less sensitive than qPCR [35].

In a pioneering advancement for feline health, the FCoV *M* gene emerged as a prime diagnostic target, captivating researchers with its pivotal role in viral assembly and pathogenicity. This highly conserved gene, the cornerstone of the virus’s structural integrity, orchestrates critical interactions with S and N proteins, fueling virion formation. Its mutations mark the sinister shift from benign FECV to deadly FIPV, making it an unparalleled marker for precise detection. By focusing on the *M* gene, cutting-edge assays—RT-PCR, nested RT-PCR, and RT-qPCR—achieve remarkable specificity, sidestepping interference from other feline viruses. This strategic choice ensures ultra-sensitive detection, transforming feline infectious peritonitis diagnosis with unmatched accuracy. These innovative molecular tools, rigorously tested on clinical samples, promise to redefine veterinary diagnostics, empowering clinicians to combat FCoV with swift, reliable precision and paving the way for enhanced disease management and surveillance in feline populations worldwide.

The novel aspect of this work lies in the pioneering development of highly specific primers targeting the FCoV *M* gene, meticulously designed to revolutionize the molecular diagnosis of FIP. These innovative primers, optimized for RT-PCR, nested RT-PCR, and RT-qPCR, demonstrate exceptional sensitivity and specificity, with RT-qPCR achieving an unprecedented detection limit of 9.14 × 10^1^ copies/µL and a 93.75% detection rate in clinical samples. This strategic focus on the *M* gene, a critical structural component integral to viral assembly and pathogenicity, enhances diagnostic precision, effectively distinguishing FCoV from other feline and related viruses without cross-reactivity. This study has integrated cutting-edge molecular techniques with rigorous optimization, thereby establishing a transformative diagnostic framework that not only elevates the accuracy and reliability of FCoV detection but also sets a new benchmark for veterinary diagnostics, offering profound implications for clinical management and epidemiological surveillance of FIP.

## 2. Results

### 2.1. Optimization of RT-PCR and Nested RT-PCR Assay

**Figure 1:**
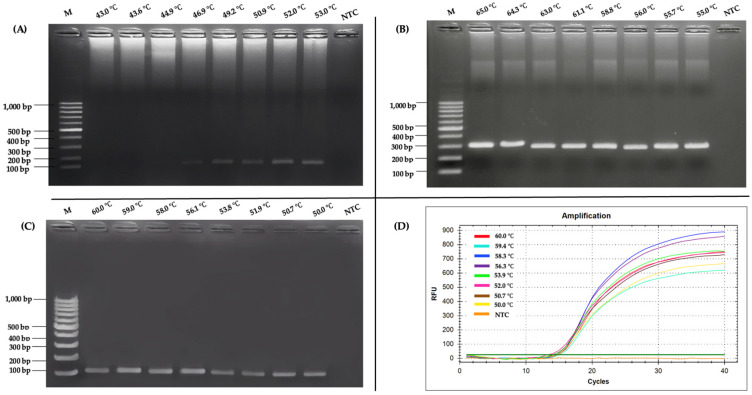
Temperature optimization. (**A**) RT-PCR; (**B**) nested RT-PCR (outer primers); (**C**) nested RT-PCR (inner primers). Agarose-gel electrophoresis (1.5%, 100 V, 30 min) shows amplicons generated at the indicated annealing-temperature gradient. Lane M, 100 bp DNA ladder (Marker III, Yeastern Biotech, New Taipei City, Taiwan); lane NTC, negative control (RNase-free water). (**D**) Evaluation of optimal annealing temperature using RT-qPCR.

The optimization of the RT-PCR assay targeting the FCoV M gene demonstrated a clear dependence on the annealing temperature to achieve efficient and specific amplification. As shown Figure 1A, the results of agarose gel electrophoresis (AGE) of RT-PCR at gradient annealing temperatures ranging from 43 °C to 53 °C revealed that amplification products became progressively more distinct and intense as the temperature approached 52.0 °C, at which the most well-defined and robust band was observed. However, at lower temperatures, the bands were faint or absent, suggesting inadequate primer annealing specificity. Conversely, temperatures above 52.0 °C led to a decrease in band intensity, likely due to excessively stringent conditions that hindered primer binding. Based on these findings, 52.0 °C was selected as the optimal annealing temperature for RT-PCR, ensuring reliable amplification of the M gene target while minimizing non-specific products, as confirmed by the absence of bands in the negative control (NTC) lane. For the nested RT-PCR assay, the optimization procedure was systematically executed in two distinct phases, each corresponding to the application of the outer and inner primer pairs. The outcomes from these phases are depicted in Figure 1B,C, respectively. In the first round, using the outer primers (N-outer MF and N-outer MR), a gradient temperature from 55 °C to 65 °C was tested, targeting a 300 bp fragment of the FCoV M gene. The AGE results indicated that 56.0 °C produced the most intense band. This temperature facilitated the efficient annealing of the outer primers to the template derived from the FIP vaccine, which functioned as the FCoV DNA template. Although 65.0 °C yielded the most intense band with the FIP vaccine template in initial AGE analysis, clinical sample testing showed suboptimal amplification with faint bands. Further optimization revealed that 56.0 °C provided consistently intense bands across clinical samples, confirming it as the optimal annealing temperature for the outer primers in nested RT-PCR, balancing specificity and sensitivity for diagnostic reliability. Additionally, the absence of any bands in the NTC lane confirmed the experiment was free from contamination. The clear 300 bp band at 56.0 °C validated this temperature as optimal for the initial amplification step, which paved the way for the subsequent nested reaction. In the second round of nested RT-PCR, the inner primers (N-inner MF and N-inner MR) were used to amplify a 125 bp product from the first-round amplicons. This step was optimized across a temperature range from 50 °C to 60 °C, as demonstrated in Figure 1C. The AGE results highlighted 56.1 °C as the optimal annealing temperature, where the band intensity was maximized, with no detectable amplification in the NTC lane. Densitometric analysis of the AGE bands confirmed that 56.1 °C yielded slightly higher intensity compared to 60 °C, 59 °C, and 58 °C, despite similar visual appearance in Figure 1C. This temperature optimized specificity and amplification efficiency, minimizing non-specific products. The selection was validated across replicate experiments, ensuring robust and reproducible results. At temperatures below 56.1 °C, fainter bands were observed, indicating suboptimal primer binding, while temperatures above 56.1 °C showed reduced yields, likely due to excessive stringency. The consistency of 56.0 °C and 56.1 °C as the optimal temperatures for the outer and inner primer sets, respectively, further emphasized the precision of the nested RT-PCR design. For the RT-qPCR assay, a gradient temperature ranging from 50 °C to 60 °C was tested, targeting a fragment of the FCoV M gene. The RT-qPCR analysis revealed that the relative fluorescent signal curve of the positive reaction appeared most rapidly at 56.3 °C (Figure 1D), which enhanced sensitivity by amplifying a shorter, more specific fragment. Subsequently, these optimized conditions were adopted for all further experiments, ensuring robust and reproducible detection of the FCoV M gene in clinical samples.

### 2.2. Specificity Analysis

**Figure 2:**
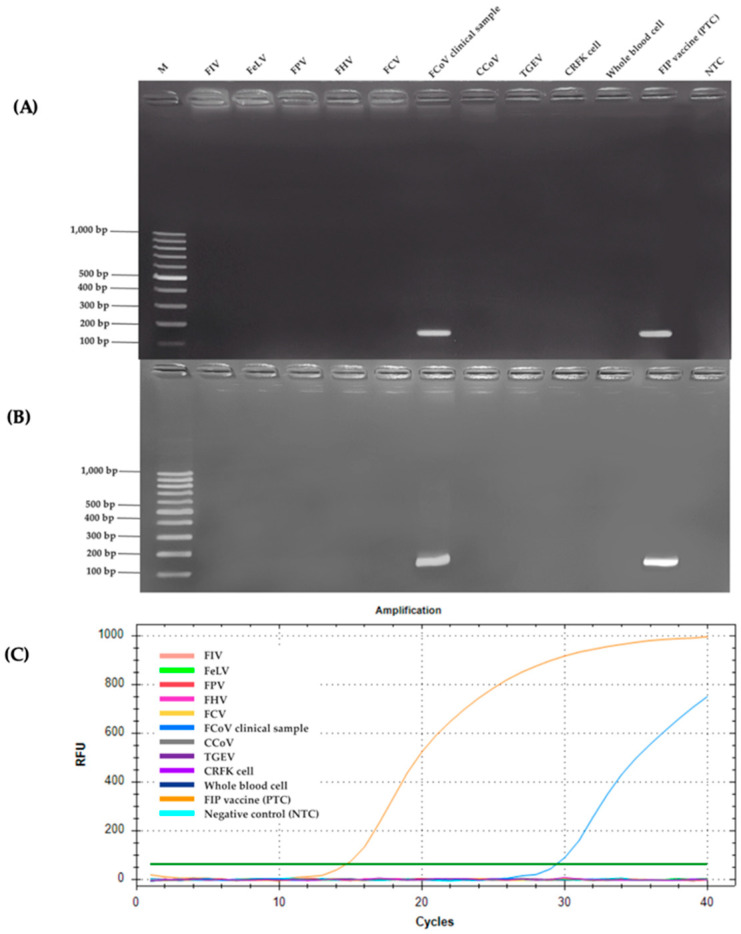
(**A**) RT-PCR, (**B**) nested RT-PCR assay. Specificity was evaluated by agarose gel electrophoresis using various feline and related viruses. Amplicons were detected only in the FCoV clinical sample and the vaccine-derived positive control. No amplification was observed in other viral targets, internal controls, or the negative control (NTC). Lane M: 100 bp DNA ladder (Yeastern Biotech, New Taipei City, Taiwan); lanes 1–10: FIV, FeLV, FPV, FHV, FCV, FCoV clinical sample, CCoV, TGEV, CRFK cells, and whole blood cells, respectively. (**C**) Evaluation of specificity using RT-qPCR assay.

**Figure 3:**
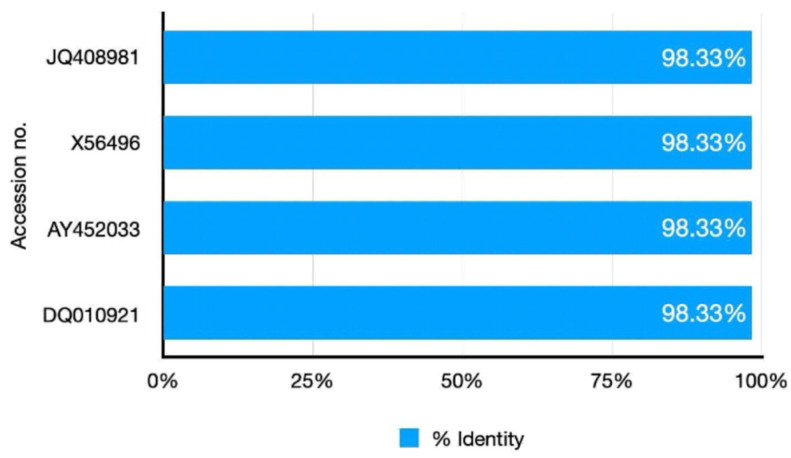
Similarity in BLAST analysis of FCoV clinical sample No. KU80 sequence.

The RT-PCR specificity analysis was conducted using AGE to assess its performance across a range of feline and related viruses, as shown in Figure 2A. Distinct bands were observed exclusively in the lanes containing the FCoV clinical sample (lane 6) and the FIP vaccine positive control (lane 11), indicating successful amplification of the target sequence. This suggested that the RT-PCR assay was highly specific to FCoV, as it did not produce detectable bands in any other tested samples. Similarly, the nested RT-PCR assay produced a comparable level of specificity when evaluated under the optimum conditions, as shown in Figure 2B. Clear amplification products were visible only in the lanes corresponding to the FCoV clinical sample (lane 6) and the FIP vaccine positive control (lane 11), with no unexpected bands appearing elsewhere on the gel. The assay’s ability to selectively amplify FCoV sequences was evident, as it consistently produced results aligned with the expected outcomes for the positive samples. This reinforced the reliability of nested RT-PCR in distinguishing FCoV from other viral sequences. In addition, the specificity of the RT-qPCR assay was assessed by evaluating its ability to detect FCoV across a range of feline and related viruses, as shown in Figure 2C. Given the context of the analysis, it could be inferred that RT-qPCR also targeted FCoV specifically, with amplification limited to the FCoV clinical sample and the FIP vaccine positive control. The absence of additional details suggested that its specificity profile aligned with the other two methods, focusing solely on the intended viral target without cross-reactivity. Across all three methods (RT-PCR, nested RT-PCR, and RT-qPCR), no amplification products were observed in lanes containing other feline viruses such as feline immunodeficiency virus (FIV), feline leukemia virus (FeLV), feline panleukopenia virus (FPV), feline herpesvirus (FHV), and feline calicivirus (FCV). Similarly, related viruses, such as canine coronavirus (CCoV) and transmissible gastroenteritis virus (TGEV), as well as internal controls including Crandell-Rees Feline Kidney (CRFK) cells and whole blood cells, showed no detectable bands. Furthermore, the negative control (NTC), consisting of RNase-free water, produced no amplification, confirming the absence of contamination. In addition, in silico analysis was conducted using reference sequences of various feline viruses: FIV (M25381), FeLV (MT129531), FPV (KP280068), FHV (NC_013590), and FCV (KP987265), as well as coronaviruses such as FCoV (DQ848678 and AY994055), CCoV (KP981644), and TGEV (NC_038861). To affirm the precision of FCoV detection, Sanger sequencing was performed on a clinical sample (KU.80), using the MF and MR primers for RT-PCR amplification, alongside the N-Inner MF and N-Inner MR primers for nested RT-PCR and RT-qPCR as sequencing primers. The resulting nucleotide sequence was carefully analyzed using BLAST version 2.16.0, a sophisticated tool for comparing genetic blueprints. The analysis revealed a remarkable 98.33% identity between the KU.80 sequence and the membrane regions of two esteemed FCoV reference strains: FIPV DF-2 (JQ408981) and FCoV FIPV 79-1146 (DQ010921), as illustrated in Figure 3. Furthermore, the sequence harmonized closely with other FCoV membrane gene segments (X56496 and AY452033), each sharing the same 98.33% identity. These findings weave a tapestry of genetic kinship, confirming that the KU.80 isolate is a faithful representative of established FCoV strains, ideal for validating detection assays. The striking similarity underscored the exquisite specificity of the primers used in RT-PCR, nested RT-PCR, and RT-qPCR, which target FCoV with unwavering accuracy, free from interference by unrelated viral sequences. This resolute specificity elevates the reliability of these assays, ensuring they serve as trusted sentinels in the diagnosis of FCoV in feline companions, thereby enriching veterinary care with confidence and care.

### 2.3. Comparison of RT-PCR, Nested RT-PCR and RT-qPCR Regarding Sensitivity

**Figure 4:**
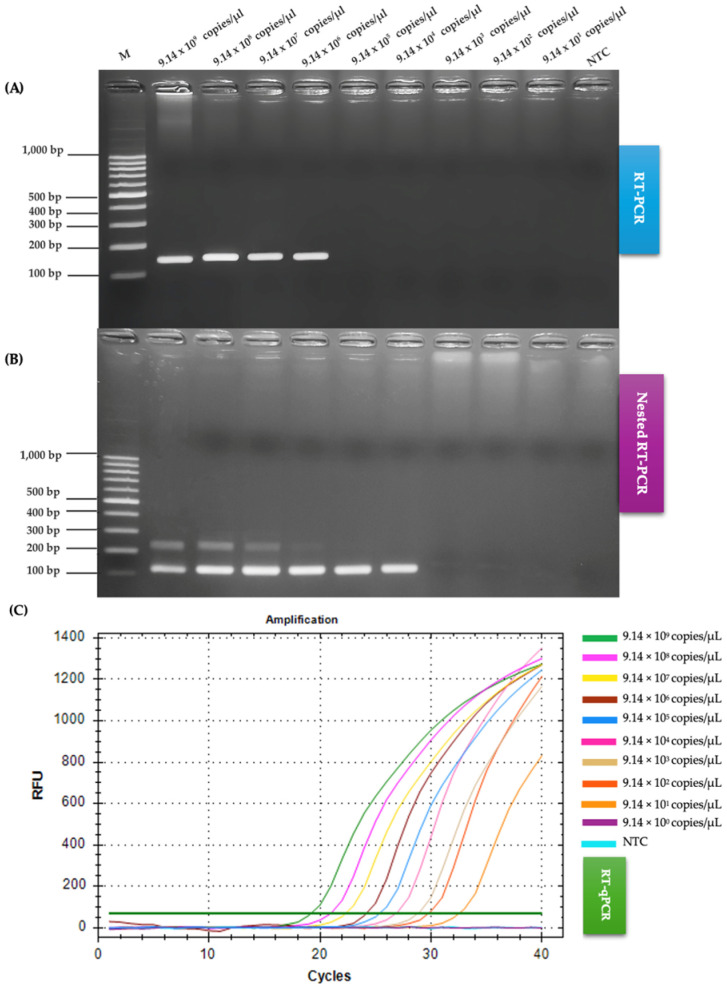
Comparison of RT-PCR, nested RT-PCR, and RT-qPCR sensitivity for FCoV detection. (**A**) RT-PCR products resolved on a 1.5% agarose gel. Lane M, 100 bp ladder; lanes 1–9, 10-fold serial dilutions from 9.14 × 10^9^ to 9.14 × 10^1^ copies µL; lane NTC, negative control. (**B**) Nested RT-PCR products analysed under identical gel conditions. (**C**) RT-qPCR standard curve derived from the same dilution series (9.14 × 10^9^ to 9.14 × 10^0^ copies µL), showing the lower detection limit of each assay.

The sensitivity of the three molecular methods—RT-PCR, nested RT-PCR, and RT-qPCR—for detecting feline coronavirus (FCoV) was compared using a 10-fold serial dilution series ranging from 9.14 × 10^9^ to 9.14 × 10^1^ copies/µL. RT-PCR had the lowest sensitivity with a detection limit of 9.14 × 10^6^ copies/µL, indicating its reliance on higher viral loads. Nested RT-PCR improved upon this, achieving a detection limit of 9.14 × 10^4^ copies/µL, owing to its two-step amplification process, making it more suitable for lower viral concentrations. Notably, RT-qPCR outperformed both, with a detection limit of 9.14 × 10^1^ copies/µL (Figure 4) demonstrating exceptional sensitivity and the ability to detect FCoV at extremely low levels. These results established RT-qPCR as the most sensitive and effective method for FCoV detection, particularly in cases with minimal viral RNA, followed by nested RT-PCR and RT-PCR.

### 2.4. Comparison of RT-PCR, Nested RT-PCR, and RT-qPCR on Clinical Samples for FCoV Detection

This study evaluated the diagnostic sensitivity of RT-PCR, nested RT-PCR, and RT-qPCR for detecting FCoV in 80 effusion fluid samples from cats suspected of having FIP. As shown in Table 1, RT-qPCR had the highest detection rate, identifying 75 positive samples (93.75%, 75/80), with only 5 negative samples. Nested RT-PCR followed, detecting 70 positive samples (87.50%, 70/80), with 10 negative samples. RT-PCR was the least sensitive, detecting 49 positive samples (61.25%, 49/80), with 31 negative samples. Using RT-qPCR as the reference standard, nested RT-PCR had a sensitivity of 93.33%, substantially higher than RT-PCR’s 65.33%. Both RT-PCR and nested RT-PCR had 100% specificity and positive predictive values (PPVs), indicating no false positives. However, their negative predictive values (NPVs) varied greatly: RT-qPCR achieved 100%, nested RT-PCR achieved 50%, and RT-PCR achieved only 16.13%. Statistical analysis confirmed RT-PCR’s detection rate was significantly lower than both nested RT-PCR and RT-qPCR (*p* < 0.05), while nested RT-PCR and RT-qPCR showed no significant difference (*p* = 1.000). These findings highlighted RT-qPCR as the most reliable method for FCoV detection due to its superior sensitivity and NPV, making it ideal for veterinary diagnostics. Nested RT-PCR is a highly sensitive alternative, particularly for laboratories lacking real-time PCR platforms, while RT-PCR’s limited sensitivity makes it less effective for ruling out FCoV infection.

To contextualize the performance of the RT-PCR, nested RT-PCR, and RT-qPCR assays developed in this study, a comparative analysis with previous studies targeting FCoV detection is presented. Earlier research has employed various molecular techniques, including RT-PCR, nested RT-PCR, RT-qPCR, and RT-LAMP, targeting different FCoV genes such as the *N*, *S*, 7b, and ORF1a/1b genes, with varying sensitivities and specificities. Table 2 summarizes key findings from selected studies compared to the current study, focusing on detection limits, sample types, and diagnostic performance.

The present study’s RT-qPCR assay, targeting the *M* gene, achieved a detection limit of 9.14 × 10^1^ copies/µL, surpassing the sensitivity of RT-qPCR assays reported by Felten et al. (2017) and Simons et al. (2005), which targeted the 3c and *M* genes, respectively, with detection limits ranging from 10^2^ to 10^3^ copies/µL [33,36]. Similarly, our nested RT-PCR assay (detection limit: 9.14 × 10^4^ copies/µL) outperformed the nested RT-PCR assay by Gamble et al. (1997), which targeted the 7b gene and achieved a higher detection limit of approximately 10^5^ copies/µL [29]. Compared to RT-LAMP assays, such as those by Rapichai et al. (2022) [34] and Khumtong et al. (2025) [35], which targeted the ORF1a/1b and *N* genes with detection limits of 1.5 × 10^5^ and 1.7 × 10^1^ copies/µL, respectively, our RT-qPCR assay demonstrated comparable or superior sensitivity. Notably, all assays in the current study maintained 100% specificity, consistent with prior studies, reinforcing the reliability of the *M* gene as a diagnostic target. The focus on effusion samples in this study aligns with clinical relevance, as effusions are highly predictive for FIP diagnosis, though some earlier studies also tested feces and blood, which may yield lower viral loads. These comparisons highlight the superior sensitivity of our RT-qPCR and nested RT-PCR assays, particularly for detecting low viral loads in clinical settings, establishing them as robust tools for FIP diagnosis.

## 3. Discussion

The FCoV is a widespread viral pathogen affecting domestic and wild cats, often presenting as a mild enteric infection, though it can escalate into the severe and typical FIP in a small percentage of cases. The *Coronaviridae* virus exists in two primary biotypes: FECV, which is ubiquitous and generally benign, while FIPV is a mutated form that triggers systemic disease through immune-mediated mechanisms [19]. Transmission occurs primarily via the fecal–oral route, with crowded environments, such as shelters, amplifying its spread. However, the transition from FECV to FIPV remains poorly understood, complicating prevention efforts. Genetic mutations and host immune responses play a major role in the shift, with factors, such as stress and co-infections, potentially increasing susceptibility to FIP development [2]. The *M* gene of FCoV is pivotal in the development of FIP, working alongside the spike protein to facilitate the transition from the benign FECV to the virulent FIPV. Mutations in this gene have been identified as key differentiators between symptomatic and asymptomatic infections. The membrane protein, essential for viral assembly and release, contains specific amino acid residues linked to FIPV virulence. These mutations enable the virus to evade the host immune response, driving severe disease outcomes. Comparative analyses of FECV and FIPV have revealed major genetic differences in the membrane gene, which may serve as diagnostic markers for detecting virulent strains in cat populations [11,12,13,14,15].

Optimization of RT-PCR, nested RT-PCR, and RT-qPCR assays targeting the FCoV *M* gene highlights the importance of the annealing temperature in achieving efficient and specific amplification. Optimal temperatures enhanced assay performance, ensuring reliable detection of FCoV in clinical samples [35,37,38]. The optimal performance of the RT-PCR assay at 52.0 °C highlighted the importance of precise temperature control in molecular diagnostics. This temperature likely provides the best balance between primer specificity and amplification efficiency, as lower temperatures produced faint or absent bands, while higher temperatures led to reduced signal intensity—both indicative of suboptimal annealing conditions [39]. The use of nested RT-PCR, with two-step optimization at 56.0 °C for outer primers and 56.1 °C for inner primers, highlighted how precise temperature control could enhance both specificity and sensitivity. This aligned with another report suggesting that nested RT-PCR is particularly effective for detecting low-abundance targets when conditions are carefully optimized [40]. The absence of bands in the NTC across all assays reinforced their reliability and freedom from contamination—a key factor in diagnostic assay validation [41]. The optimal annealing temperature of 56.3 °C for RT-qPCR, determined through real-time fluorescence, underscored its efficiency in rapidly amplifying short, specific fragments and supported recent findings in veterinary diagnostics that highlighted RT-qPCR’s superior sensitivity and speed [41]. Overall, these findings underscored the importance of systematic optimization, as slight temperature deviations can seriously impact performance, aligning with modern standards for PCR-based pathogen detection [39]. The optimized conditions of these assays contribute to improved reproducibility and specificity, reinforcing their value in FCoV detection.

High specificity is crucial for ensuring diagnostic accuracy, particularly in clinical settings where co-infections or overlapping symptoms can complicate disease identification [42]. The specificity analyses of RT-PCR, nested RT-PCR, and RT-qPCR assays for detecting FCoV demonstrated their robust performance in selectively amplifying FCoV sequences without cross-reactivity to other feline or related viruses. All three methods consistently produced amplification products only in the FCoV clinical sample and the FIP vaccine positive control, with no detectable signals in lanes containing feline immunodeficiency virus (FIV), feline leukemia virus (FeLV), feline panleukopenia virus (FPV), feline herpesvirus (FHV), feline calicivirus (FCV), canine coronavirus (CCoV), transmissible gastroenteritis virus (TGEV), or non-target controls such as CRFK cells, whole blood cells, and the NTC. Other research has reported promising approaches in differentiating FCoV from other coronaviruses, which is crucial for improving diagnostic accuracy. One notable method involves RT-PCR assays that specifically target the FCoV *M* gene. This gene has been identified as a reliable target due to its ability to enhance specificity, thereby minimizing the risk of false-positive results in clinical diagnostics. Such findings support the use of gene-specific assays in veterinary virology to improve diagnostic outcomes [43]. The in silico analysis further supported these findings, showing that the primer sets had strong binding affinity exclusively to FCoV reference sequences (DQ848678 and AY994055), consistent with modern bioinformatics approaches to primer design [44]. This computational validation supported the laboratory findings and aligned with another study that emphasized the importance of in silico analysis in primer design to ensure specificity [45]. These results underscored the reliability of these assays for accurate FCoV detection, a critical factor in managing FIP, where precise diagnosis is essential due to its fatal prognosis [19]. The comparable specificity across RT-PCR, nested RT-PCR, and RT-qPCR highlighted their suitability for clinical application, though their practical utility may differ based on procedural complexity and diagnostic needs. Nested RT-PCR, with its two-step amplification, had improved sensitivity compared to RT-PCR while maintaining equivalent specificity, as reported [46], making it particularly useful for detecting low-viral-load samples. RT-qPCR, inferred to maintain similar specificity, provides the added advantage of real-time monitoring, which has been praised for its speed and precision in recent veterinary diagnostics [47]. The absence of amplification in non-target samples across all methods was consistent with established specificity criteria [48], which emphasize the need to prevent cross-reactivity with related coronaviruses such as CCoV and TGEV in FCoV assays.

Several genes have been used as targets for feline coronavirus (FCoV) detection, including the 7b, *N*, *S*, and ORF1a/1b genes. Among these, the 7b gene has been widely employed in conventional RT-PCR assays due to its presence in both FECV and FIPV strains; however, its sensitivity is often lower in clinical samples, particularly during early infection stages [25]. The *S* gene, which encodes the spike protein, has shown specificity for FIPV-associated mutations but exhibits high sequence variability, which can compromise primer binding and reduce assay reliability across different strains [11,26]. The *N* gene has also been utilized in RT-PCR and qPCR formats with moderate success, though studies have reported inconsistent amplification results in field samples [33]. On the other hand, the *M* gene, encoding the membrane protein, is more conserved among FCoV strains and has demonstrated superior sensitivity and detection limits in both conventional and real-time PCR assays [16]. Due to its genetic stability and high transcription levels during viral replication, the *M* gene has been considered a reliable and efficient target for molecular diagnosis of FCoV [17].

The sensitivity of molecular diagnostic methods for detecting FCoV has been a critical focus in veterinary virology research [34,35,49]. Comparative studies have revealed large differences among RT-PCR, nested RT-PCR, and RT-qPCR techniques. The sensitivity of molecular diagnostic methods for detecting FCoV has been a pivotal topic in veterinary virology, with techniques, such as RT-PCR, nested RT-PCR, and RT-qPCR, showcasing distinct strengths and limitations. Comparative studies have highlighted that RT-PCR, while reliable and straightforward, lags in sensitivity with a detection limit of 9.14 × 10^6^ copies/µL, rendering it less effective for samples with low viral loads. In contrast, nested RT-PCR, with its two-step amplification, yielded sensitivity to 9.14 × 10^4^ copies/µL, making it a valuable tool for detecting FCoV in cases with reduced viral RNA, such as chronic infections or effusion fluids from FIP cases. Notably, RT-qPCR had the highest sensitivity, detecting as low as 9.14 × 10^1^ copies/µL, making it the most effective technique currently available for clinical FCoV detection, particularly in effusive or chronic cases. This remarkable sensitivity, driven by real-time quantification and optimized probe design, makes RT-qPCR particularly suited for identifying minimal viral RNA, a critical factor in managing asymptomatic carriers and enhancing epidemiological monitoring. Innovations such as the one-step triplex RT-qPCR, capable of detecting FCoV, FPV, and FeLV simultaneously, highlight the potential of multiplexing to streamline diagnostics [50]. This approach enhances specificity and efficiency by consolidating multiple targets into a single reaction tube. These advancements reflect the ongoing effort to refine molecular tools, addressing challenges such as low viral loads and contributing to improved disease management in feline populations [51]. 

Considering their practical use, all three methods—RT-PCR, nested RT-PCR, and RT-qPCR—were effective for detecting FCoV in effusion fluids. However, nested RT-PCR and RT-qPCR were slightly more accurate in detecting the virus than RT-PCR [29]. This aligned with other research, yet the statistical analysis in the current study revealed no significant difference in diagnostic yield (*p* = 1.000). Nested RT-PCR has demonstrated effectiveness in detecting low viral loads in various infections. For example, a study on rotavirus A developed a sensitive nested RT-PCR assay capable of detecting viral loads as low as 6.2 copies per reaction, highlighting its utility in low-viral-load scenarios [52]. Similarly, research on human bocavirus (HBoV) found that real-time PCR assays could detect as few as 10 copies of HBoV DNA, offering a two-log increase in sensitivity over conventional PCR methods [53]. These findings have underscored the superior analytical sensitivity of RT-qPCR for low-copy targets, making it a valuable tool in clinical diagnostics. RT-PCR maintains its relevance in resource-limited settings by offering a balance of high sensitivity and specificity with reduced cost and complexity. The selection among RT-qPCR, nested RT-PCR, and RT-PCR for FCoV detection is influenced by specific diagnostic needs and available resources. RT-qPCR is renowned for its precision and multiplexing capabilities, allowing simultaneous detection of multiple pathogens, which enhances diagnostic efficiency in veterinary settings. For example, multiplex one-step RT-qPCR assays have been developed to detect various enteric viruses in dogs and cats, including FCoV, demonstrating the method’s applicability in comprehensive diagnostic panels [54]. Nested RT-PCR serves as a robust alternative, particularly valuable in cases with low viral loads where enhanced sensitivity is required. One study has shown that nested RT-PCR could effectively detect low viral loads of viruses, such as SARS-CoV-2 in animal samples, highlighting its utility in challenging diagnostic scenarios [55]. However, the efficacy of these PCR-based diagnostics is heavily dependent on factors including RNA quality and reaction conditions. Research has indicated that inhibitors present in fecal samples could affect the quantification of viral RNA, underscoring the necessity for meticulous sample preparation and validation of assay conditions [20]. Collectively, while RT-qPCR offers leading sensitivity, the complementary strengths of nested RT-PCR and RT-PCR provide a versatile diagnostic toolkit for managing FCoV across diverse veterinary scenarios.

This study establishes a significant advancement in FCoV detection by demonstrating the superior sensitivity and specificity of RT-qPCR and nested RT-PCR assays targeting the *M* gene, particularly for effusion-based samples. Compared to earlier studies, such as those by Felten et al. (2017) [36] and Gamble et al. (1997) [29], which reported detection limits of 10^2^–10^3^ copies/µL and 10^3^ copies/µL, respectively, our RT-qPCR assay achieved a detection limit of 9.14 × 10^1^ copies/µL, detecting 93.75% of clinical samples, while nested RT-PCR reached 9.14 × 10^4^ copies/µL with an 87.50% detection rate. Both assays maintained 100% specificity, showing no cross-reactivity with other feline viruses, unlike some prior methods that reported variable specificity. The focus on the conserved *M* gene and optimized assay conditions enhances diagnostic accuracy for FIP in effusion samples, which are highly predictive for clinical diagnosis. These findings position our assays as cutting-edge tools, surpassing previous molecular methods by offering unmatched sensitivity and reliability, thus addressing critical diagnostic needs in veterinary practice and paving the way for improved FIP management and surveillance.

The current study has demonstrated that nested RT-PCR and RT-qPCR outperformed conventional RT-PCR in detecting FCoV in feline effusion samples, with RT-qPCR having the highest detection rate (93.75%), followed closely by nested RT-PCR (91.25%). These findings are consistent with other research, where nested RT-PCR has shown superior sensitivity in detecting viral pathogens, especially in low-viral-load samples [56,57]. Based on the current findings, the comparable performance between nested RT-PCR and RT-qPCR supports the use of nested RT-PCR as a reliable, cost-effective alternative when real-time PCR is not available. Notably, both methods significantly outperformed RT-PCR, aligning with studies that highlighted the limitations of single-step RT-PCR in samples with low RNA concentrations [58]. Viral load plays a critical role in detection accuracy. Low viral loads can lead to false negatives, particularly with less sensitive assays, such as, conventional RT-PCR, as reported [59], where it was observed that FCoV RNA may be undetectable in effusions with minimal viral replication. Thus, assays with higher analytical sensitivity, such as nested RT-PCR and RT-qPCR, are essential for reliable FCoV detection. In summary, nested RT-PCR offers diagnostic sensitivity nearly equivalent to RT-qPCR and superior to RT-PCR, particularly in samples with a low viral load, making it a valuable tool for FIP diagnosis in clinical settings.

Despite the exemplary performance of RT-PCR, nested RT-PCR, and RT-qPCR assays in detecting FCoV with outstanding sensitivity and specificity, the current study was not without limitations. The evaluation was predominantly restricted to effusion samples from cats suspected of FIP, which may constrain the applicability of the findings to other sample types, such as blood, feces, or tissue biopsies, where viral loads and matrix effects could differ notably. Furthermore, the study targeted a specific subset of FCoV strains prevalent in the sampled population, leaving uncertainties regarding the efficacy assays against diverse or emergent viral genotypes. The resource-intensive nature of RT-qPCR, despite its superior sensitivity, presents practical challenges for adoption in resource-limited veterinary settings, highlighting the need for cost-effective alternatives. To address this, the loop-mediated isothermal amplification (LAMP) technique, previously developed by our group for FCoV detection, offers a promising solution. RT-LAMP [34,35], with its rapid, isothermal amplification and minimal equipment requirements, mitigates the resource demands of RT-qPCR while maintaining high sensitivity and specificity, as demonstrated in another studies targeting the FCoV nucleocapsid gene [35]. These limitations should inspire future research to validate LAMP and nested RT-PCR across broader clinical and epidemiological contexts, enhancing the adaptability of these innovative diagnostic tools and strengthening global FCoV surveillance efforts.

## 4. Materials and Methods

### 4.1. Ethics Statement

The study obtained ethical approval from the Institutional Animal Care and Use Committee of Kasetsart University, Bangkok, Thailand (Protocol code ACKU67-VET-123, Date 25 November 2024) and was conducted according to the guidelines of the Declaration of Helsinki. Additionally, sample collection was authorized by each cat’s owner.

### 4.2. Collection of Clinical Samples

In this study, 80 effusion fluid samples were meticulously collected from cats suspected of having feline infectious peritonitis (FIP) at the Kasetsart Veterinary Teaching Hospital in Bangkok, Thailand. These samples were gathered from the thoracic and/or abdominal cavities. To ensure diagnostic accuracy, a stringent inclusion protocol was implemented, mandating that each sample meet at least four FIP-associated criteria. These criteria encompassed abnormal serum biochemical and hematological profiles, clinical signs such as abdominal distension or pleural effusion, radiographic or ultrasonographic evidence of fluid accumulation, a positive Rivalta’s test, and molecular confirmation through PCR detecting FCoV. This standardized approach to sample collection was designed to reduce variability and ensure that only clinically relevant samples were analyzed.

### 4.3. RNA Extraction

Viral RNA was extracted using the E.Z.N.A. Viral RNA Kit (Omega Bio-Tek; mega Bio-tek, Norcross, GA, USA) following the manufacturer’s protocol, and extracted RNA was stored at −80 °C to preserve integrity and stability. Then, the extracted RNA was used as a template for RT-PCR optimization. To synthesize complementary DNA (cDNA) for FCoV, RNA obtained from effusion fluid supernatants was used as a template in combination with the RevertAid First Strand cDNA Synthesis Kit (Thermo Scientific; USA). The cDNA samples were analyzed for the *M* gene using RT-PCR primers to confirm the presence of FCoV. The reverse transcription reaction was carried out in a 10 μL reaction volume using the RevertAid First Strand cDNA Synthesis Kit (Thermo Scientific; Wilmington, NC, USA), with the following components: 2 μL of RNA sample, 0.5 μL of Oligo (dT) primer, 3.5 μL of RNase-free water, 2 μL of 5X reaction buffer, 0.5 μL of RiboLock RNase Inhibitor, 1 μL of dNTP Mix, and 0.5 μL of RevertAid M-MuLV Reverse Transcriptase. The reaction was performed using a thermal cycler (Bio-Rad, Hercules, CA, USA) at 42 °C for 60 min, followed by enzyme inactivation at 70 °C for 5 min. The final RNA concentration was measured using a NanoDrop spectrophotometer (Thermo Scientific; Wilmington, NC, USA) to ensure sample quality before further analysis.

### 4.4. RT-PCR and Nested RT-PCR Primer Design

**Figure 5:**
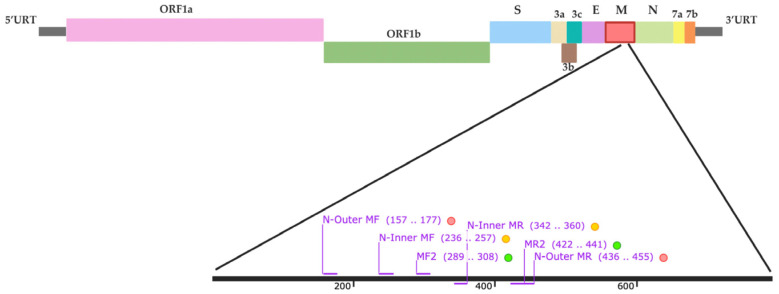
Schematic representation of the FCoV genome, comprising the 5′ untranslated region (UTR), ORF1a/1b, spike (S), ORF3a, ORF3b, ORF3c, envelope (E), membrane (M), nucleocapsid (N), ORF7a, ORF7b, and the 3′ UTR. Locations of the RT-PCR outer primers (MF, MR) and nested RT-PCR/RT-qPCR inner primers (N-Inner MF, N-Inner MR) within the M gene are indicated.

The RT-PCR primers were designed based on the nucleotide sequence of the M region of the FCoV genome. Reference sequences were obtained from the GenBank database (https://www.ncbi.nlm.nih.gov/nucleotide/ (accessed on 19 June 2025)). accession numbers (AB781788, AB781789, AY994055, DQ286389, GQ152141, JN634064, and JQ404410) that were used to design RT-PCR primers using the SnapGene version 8.0.1 software. www.snapgene.com (accessed on 9 April 2024). The specificity of the designed primers was evaluated using Primer-BLAST by comparing them against sequences from feline infectious peritonitis virus (AY994055), feline immunodeficiency virus (M25381), feline leukemia virus (MT129531), feline herpesvirus (NC_013590), feline calicivirus SH (KP987265), and feline panleukopenia virus (MG924893). The primer was synthesized by Macrogen (Seoul, Republic of Korea), and their sequences are provided in Table 2. The information for the primer sets used in this study is presented in Figure 5.

### 4.5. RT-PCR, Nested RT-PCR, and Optimization

Various reaction parameters were tested to optimize the RT-PCR conditions for enhancing genetic material amplification. The RT-PCR reaction was carried out in a total volume of 15 µL, consisting of 7.5 µL of 2X AccuStart™ II GelTrack™ PCR SuperMix (Quantabio; Beverly, MA, USA), 0.9 µL (0.6 µM) of each primer (MF2 and MR2), 1 µL of cDNA from the FIP vaccine, and 3.7 µL of RNase-free water. Negative controls were prepared using RNase-free water. The FIP vaccine (Primucell^®®^ FIP, Zoetis Inc., Lincoln, NE, USA), a modified-live, temperature-sensitive vaccine containing the attenuated FCoV strain DF2-ts, was used as a positive control. The vaccine was imported by Zoetis (Thailand) Limited, Bangkok, Thailand (Reg. No. 1F 39/56 (B)). Amplification was performed using a Thermocycler (Bio-Rad; Hercules, CA, USA) under the cycling conditions of an initial denaturation at 94 °C for 5 min, followed by 30 cycles of denaturation at 94 °C for 1 min, annealing at a gradient temperature of 43 °C–53 °C for 45 s, and an extension at 72 °C for 1 min, with a final extension at 72 °C for 5 min. For nested RT-PCR, the first round of amplification targeted the FCoV *M* gene using the N-outer MF and N-outer MR primers (Table 2), yielding a 300 bp DNA fragment. The reaction was conducted for 30 cycles under the same conditions as RT-PCR, except for the annealing temperature, which was set to 55–65 °C. Then, the resulting PCR amplicons were used as templates for the second round of nested RT-PCR, which utilized the N-inner MF and N-inner MR primers (Table 2) to generate a 125 bp DNA product. The second round of nested RT-PCR was performed by amplifying 1 µL of the first-stage PCR product in a 15 µL reaction mix, which contained the same components as the initial PCR, except that N-inner MF and N-inner MR primers were used instead of the outer primer set. This reaction was conducted for 30 cycles, with an annealing gradient temperature of 50–60 °C. Following amplification, PCR products were mixed with loading dye and analyzed by agarose gel electrophoresis. A 1.5% (*w*/*v*) agarose gel was prepared by dissolving 0.9 g of agarose in 60 mL of 1× TAE buffer (Tris-acetate-EDTA) and heating until fully dissolved. The solution was cooled to ~50 °C, poured into a gel tray with a comb, and allowed to solidify at room temperature for 30 min.

The gel was placed in an electrophoresis chamber containing 1× TAE buffer. DNA samples (5 µL) were mixed with 1 µL of Safe-Green™ loading dye and loaded into the wells. A 100 bp DNA ladder was used as a molecular weight marker. Electrophoresis was carried out at 100 V for 30 min. DNA bands were visualized using a BluPAD LED transilluminator (BIO-HELIX, New Taipei City, Taiwan).

For reverse transcription quantitative PCR (RT-qPCR) optimization, genetic alterations were detected using RT-qPCR with the 2X Maxima SYBR Green qPCR Master Mix commercial kit (Thermo Scientific; Wilmington, NC, USA). RNase-free water served as a negative control. Primers, derived from the nested RT-PCR primers (N-Inner MF and N-Inner MR) for the study in Table 3, were designed to produce a 125 bp DNA fragment. The reaction mixture for each sample consisted of 1 µL of cDNA, 0.9 µL of each primer, 7.5 µL of Master Mix, and 4.7 µL of RNase-free water. Reactions were performed in optical 96-well plates, with each sample analyzed in triplicate to ensure experimental and technical reliability. Amplification cycles consisted of denaturation at 95 °C for 30 s, an annealing gradient temperature of 50–60 °C, followed by 40 cycles and an extension at 72 °C for 1 min, followed by a final extension at 72 °C for 5 min. The amplification products were monitored at each elongation step using a CFX96 Touch Real-Time PCR Detection System (Bio-Rad; Hercules, CA, USA).

### 4.6. Specificity Analysis

To assess the specificity of the RT-PCR, nested RT-PCR, and RT-qPCR reactions, viral genomic material (DNA or RNA) was extracted from clinical samples infected with various feline viruses, consisting of FCoV, FIV, FeLV, FHV, FCV, FPLV, CRFK cell, whole blood cell, CCoV, and TGEV. Then, these samples were amplified using RT-PCR primers under optimized reaction conditions. RNase-free water was included as a negative control (NTC), while the FIP vaccine served as a positive control. The MF and MR primers for RT-PCR amplification, and the N-Inner MF and N-Inner MR primers for the nested RT-PCR and RT-qPCR methods were used as sequencing primers (Table 2).

### 4.7. Comparison of RT-PCR, Nested RT-PCR, and RT-qPCR on Sensitivity for FCoV Detection

The comparative sensitivity of the RT-PCR assay was evaluated in comparison with nested RT-PCR and RT-qPCR by determining their values for the limit of detection. A 10-fold serial dilution of the FIP vaccine was prepared at various concentrations and used as a template in the RT-PCR, nested RT-PCR, and RT-qPCR reactions. RNase-free water served as a negative control. Amplification was performed using a real-time PCR machine, with each experiment conducted in triplicate to ensure reproducibility. The results were automatically recorded by the instrument and subsequently analyzed. To determine the detection limit in terms of viral copy number, the DNA concentration was calculated using the following equation [39,42]: number of copies = (DNA concentration (ng/µL) × [6.022 × 10^23^])/(length of nucleotide (bp) × [1 × 10^9^] × 650).

### 4.8. Detection of FCoV in Clinical Samples Using RT- PCR Assay

To evaluate the applicability of the RT-PCR procedure in a clinical setting, 80 clinical specimens (effusion fluid from cats suspected of FCoV infection) were collected between 2022 and 2024 at the Kasetsart Veterinary Teaching Hospital, Bangkok, Thailand. For FCoV RNA preparation, body fluid samples were vortexed vigorously, aliquoted into 300 μL portions, and mixed with 1X phosphate-buffered saline (PBS), pH 8.3, at a 1:3 ratio. Then, the mixture was centrifuged at 15,000 rpm for 5 min, and the supernatant was extracted for RNA using the E.Z.N.A. Viral RNA Kit (Omega Bio-Tek; Norcross, GA, USA). All extracted RNA samples were stored at −80°C to preserve integrity and stability until further analysis. Subsequently, the cDNA synthesized from these RNA samples was used as a template for RT-PCR analysis under the optimized reaction conditions. The FIP vaccine was used as a positive control, while RNase-free water served as a negative control (NTC). To confirm amplification, the RT-PCR products were subjected to electrophoresis on a 1.5% (*w/v*) agarose gel, followed by loading dye staining. Positive reactions had a distinctive DNA banding pattern, confirming successful amplification.

### 4.9. Statistical Analysis

A chi-squared test was performed to evaluate statistical differences among the diagnostic assays and used to compare categorical outcomes across methods. A *p*-value less than 0.05 was considered indicative of statistical significance. All analyses were carried out using the SPSS Statistics version 26 software (SPSS Inc.; Chicago, IL, USA). Diagnostic performance metrics consisting of sensitivity, specificity, positive predictive value (PPV), and negative predictive value (NPV) were calculated for nested RT-PCR using two-by-two contingency tables, with the RT-qPCR results serving as the reference standard.

## 5. Conclusions

This study has enhanced FCoV detection through the optimization and comparison of RT-PCR, nested RT-PCR, and RT-qPCR assays targeting the *M* gene, with the results demonstrating remarkable specificity and sensitivity. The findings highlighted the superior capacity of RT-qPCR to identify low viral loads, delivering exceptional precision for diagnosing feline infectious peritonitis in clinical environments. Nested RT-PCR stood out as a robust, cost-effective alternative, performing comparably to RT-qPCR, which could be especially useful in resource-constrained settings. The novel primers developed for the *M* gene improved diagnostic accuracy, representing a considerable advancement in veterinary virology. These assays should facilitate early detection and open avenues for broader applications, including monitoring viral evolution and evaluating treatment outcomes. The results should encourage further research to validate these tools across varied sample types and viral strains, bolstering worldwide feline coronavirus surveillance. By merging innovative molecular techniques with practical solutions, this work should equip veterinarians with dependable, versatile tools, revolutionizing feline infectious peritonitis management and contributing to efforts against emerging coronaviruses.

## Figures and Tables

**Table 1:** Comparison of RT-PCR, nested RT-PCR, and RT-qPCR assays for detecting FCoV in 80 clinical samples.

Detection Method	Positive	Negative	Total
**RT-PCR**	49	31	80
**Nested RT-PCR**	70	10	80
**RT-qPCR**	75	5	80

RT-PCR positive rate of FCoV: 49/80 = 61.25%; nested RT-PCR positive rate of FCoV: 70/80 = 87.50%; RT-qPCR positive rate of FCoV: 75/80 = 93.75%.

**Table 2:** Comparison of the current study with previous studies on FCoV detection.

Method	Target Gene	Sample Type	Detection Limit (copies/µL)	Sensitivity (%)	Specificity (%)	Ref.
RT-qPCR	3c	Effusion, serum/plasma	~10^2^–10^3^	90	96	[33]
RT-qPCR	*M*	Effusion, feces	~10^3^	85	100	[36]
Nested RT-PCR	7b	Effusion, tissue	~10^5^	80	100	[29]
RT-LAMP	ORF1a/1b	Effusion	1.5 × 10^5^	100	100	[34]
RT-LAMP-XO	*N*	Effusion	1.7 × 10^1^	100	100	[35]
RT-qPCR	*M*	Effusion	9.14 × 10^1^	93.75	100	This study
Nested RT-PCR	*M*	Effusion	9.14 × 10^4^	87.50	100	This study
RT-PCR	*M*	Effusion	9.14 × 10^9^	61.25	100	This study

**Table 3:** List of primers used in this study for FCoV M gene amplification.

Primer Name	Sequence (5′→3′)	Ref.
MF2 ^a^	GCDCTTACGATTTTTAATGC	The current study
MR2 ^a^	CCACMAWGABTTRGTHCTTC	
N-Inner MF ^b,c^	TCGTTKATGGCATTAAAATGC	
N-Inner MR ^b,c^	TGCAACACTAAAGCCGAAC	
N-Outer MF ^b^	AACTGGAACTTCAGCTGGTCT	
N-Outer MR ^b^	TCAGGRTTRAAAGACCACCA	

Degenerated bases were used according to IUPAC nomenclature: R (A/G), W (A/T), Y (C/T), M (A/C), K (G/T), and S (C/G). ^a^ RT-PCR; ^b^ nested RT-PCR and ^c^ RT-qPCR.

## Data Availability

The data presented in this study are available within the article. Raw data supporting this study are available from the corresponding author.
